# Managing Anti-Nutritional Factors in Plant-Based Feeds: Implications for Herbivore Nutrition and Production

**DOI:** 10.3390/metabo16070456

**Published:** 2026-06-29

**Authors:** Mingxia Han, Xiaoyu Liu, Yi Guo, Qingyu Xu, Lin Wei, Jinjin Wei, Muhammad Zahoor Khan, Changfa Wang, Zhenwei Zhang

**Affiliations:** College of Agriculture and Biology, Liaocheng University, Liaocheng 252000, China

**Keywords:** anti-nutritional factors, herbivore nutrition, tannins and phytates, rumen microbial metabolism, nutrient bioavailability

## Abstract

Anti-nutritional factors (ANFs) in terrestrial plant feeds constrain efficient herbivore production, an issue intensified by rising feed costs and growing demand for animal products. Unlike previous reviews that focus on single ANFs or feed types, this review provides an integrated, cross-species framework linking ANF chemistry, rumen microbial interactions, and mitigation strategies. It examines major ANF classes—tannins, phytates, saponins, oxalates, protease inhibitors, lectins, glucosinolates, and gossypol—and their distribution and biochemical modes of action. Mechanistic pathways are grouped into digestive effects (reduced palatability and enzyme inhibition), microbial effects (altered rumen microbiota and fermentation), metabolic effects (impaired absorption), and mineral interactions (nutrient complexation and chelation). Species-specific responses are evaluated, emphasizing the partial detoxification capacity of the rumen microbiome and the dose-dependent nature of ANF effects. Mitigation strategies—physical, chemical, microbial, enzymatic, probiotic, and genetic—are critically assessed for efficacy, scalability, and sustainability. Emerging metabolomic and metagenomic evidence shows that certain ANFs confer functional benefits at controlled doses; for example, tannins improve nitrogen retention, saponins reduce methane, and phytic acid scavenges free radicals. This synthesis supports strategic management rather than complete elimination, informing safe and sustainable use of terrestrial feeds under evolving food-security and environmental challenges.

## 1. Introduction

Terrestrial plant-based feeds—including forages, crop residues, and agro-industrial by-products—constitute the nutritional foundation of herbivorous livestock production systems worldwide [1,2,3]. Their widespread adoption reflects inherent advantages of availability, cost-effectiveness, and alignment with sustainable agricultural practices [1,4,5]. However, feed raw material costs keep rising and account for a large share of overall production costs. Combined with the growing global demand for animal protein [6], has necessitated the strategic incorporation of alternative and unconventional feed resources into herbivore diets. However, alongside essential nutrients such as proteins, carbohydrates, minerals, and vitamins, many terrestrial plants also contain naturally occurring compounds that can interfere with nutrient utilization and animal performance [7,8,9]. This shift has brought renewed attention to these naturally occurring compounds that can compromise nutrient utilization and animal performance—collectively termed anti-nutritional factors (ANFs).

ANFs are bioactive substances generated through normal plant metabolism that interfere with feed intake, digestion, absorption, or metabolic utilization of nutrients, thereby negatively affecting animal productivity and health [10,11]. Beyond their direct physiological effects, ANFs also influence the extent to which plant resources can be safely and efficiently used in diets for both humans and livestock [12]. These compounds—also referred to as antinutrients, antinutritive factors, or plant secondary metabolites [13,14]—are ubiquitous across terrestrial plant species [15] and exhibit considerable variation in concentration among plant tissues, developmental stages, and environmental conditions [16]. The major classes of ANFs in terrestrial feeds include tannins, phytates, oxalates, saponins, alkaloids, protease inhibitors [17,18]. From an evolutionary perspective, many ANFs function as chemical defense mechanisms against herbivory, pathogens, and environmental stress. In livestock nutrition, however, these same compounds may reduce palatability, inhibit digestive enzymes, chelate essential minerals, or exert direct toxic effects when present above threshold concentrations [9,14,17]. Their physiological impacts are multifaceted: some impair protein digestibility through enzyme inhibition, others form insoluble complexes with minerals and reduce bioavailability, while certain compounds compromise intestinal integrity and immune function [9,19]. Critically, the magnitude and nature of these effects are strongly influenced by animal species and digestive physiology. Ruminants, equipped with a specialized microbial ecosystem capable of degrading or detoxifying certain ANFs during ruminal fermentation, often exhibit greater tolerance than non-ruminant herbivores, which lack extensive microbial fermentation capacity and consequently demonstrate heightened sensitivity [14,20].

Contemporary challenges in livestock production—including climate change, feed scarcity, and economic pressures—have accelerated the utilization of non-conventional forages and by-products, many containing elevated or inadequately characterized ANF profiles [21]. Concurrently, advances in analytical chemistry, rumen microbiology, and molecular biology have enhanced our capacity to identify, quantify, and elucidate the mechanisms through which ANFs influence animal systems. Despite this progress, significant knowledge gaps persist regarding species-specific responses, synergistic interactions among multiple ANFs, and the efficacy of mitigation strategies under diverse production conditions [22].

As herbivores become increasingly dependent on terrestrial plant feeds, systematic and comprehensive research on ANFs is urgently needed [23]. These substances widely hinder nutrient utilization, impair absorption and animal health, and significantly affect breeding economic outcomes. Current studies mostly concentrate on individual ANFs or single feed types, failing to conduct systematic comparisons across diverse feed resources and herbivore species, explore integrated mechanisms among ANFs, rumen microbiota, and host metabolism, or recognize the dose-dependent beneficial effects of certain ANFs. Notably, Tan et al. found strong coordination between gut microbiota and the liver in animals consuming toxic plants: when ANFs enter the body, both systems work synergistically to mount adaptive responses [24].

To address these deficiencies, this review provides a narrative overview of ANFs in major terrestrial feeds, covering their major classes and sources, multi-pathway mechanisms of action, species-specific responses, and dual roles under appropriate doses [25]. It further evaluates practical and sustainable mitigation strategies, with the aim of informing evidence-based feeding practices that optimize nutrient utilization, safeguard animal health, and enhance the sustainability of herbivorous livestock production systems [26].

## 2. Materials and Methods

We conducted a comprehensive narrative review encompassing studies that investigate the occurrence, chemical characteristics, physiological mechanisms, and mitigation strategies of ANFs in terrestrial plant-based feeds, as well as their impacts on nutrient utilization and production performance in herbivores. The identification of relevant articles was accomplished through rigorous search methodologies using reputable academic databases, including Web of Science Core Collection, Scopus, PubMed, CAB Abstracts, and Google Scholar.

The search strategy was thoroughly designed, employing key terms such as “anti-nutritional factors”, “ANFs”, “herbivore nutrition”, “ruminant”, “non-ruminant”, “tannins”, “phytate”, “gossypol”, “glucosinolates”, “protease inhibitors”, “lectins”, “forage”, “oilseed meal”, “cereal”, “crop residue”, “feed utilization”, “rumen microbiota”, and “mitigation strategies” to retrieve relevant literature. Our focus was primarily directed toward sourcing data published in the English language and featured in highly regarded peer-reviewed journals. Specifically, we prioritized articles published from the year 2000 to 2025 to ensure the inclusion of contemporary research findings.

To maintain the highest standards of academic rigor, we deliberately excluded conference abstracts, letters, editorials, non-peer-reviewed sources, and duplicate publications. Studies focusing solely on human nutrition, aquaculture, or non-herbivorous animals were also excluded. This stringent selection process ensured that the information discussed in this review is grounded in credible and peer-reviewed scientific literature, contributing to the reliability and integrity of our analysis.

To translate this evidence base into a structured analysis, the following section first characterizes the major terrestrial feed resources used in herbivore production—forages, cereals, oilseed meals, and agro-industrial by-products—together with the anti-nutritional factors associated with each, thereby establishing the compositional foundation for the mechanistic and metabolic discussion that follows.

## 3. Terrestrial Feed Resources for Herbivores

**Figure 1:**
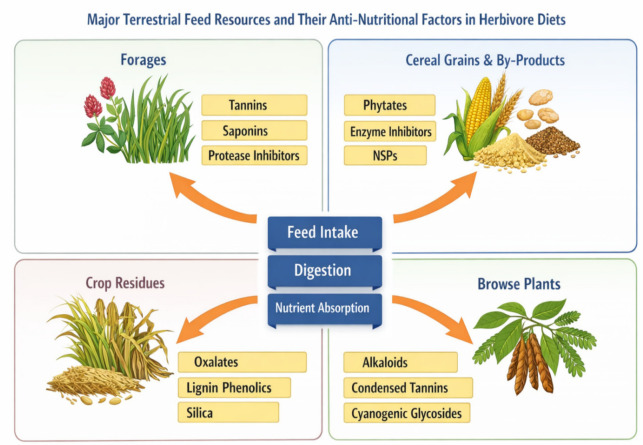
A schematic overview showing major terrestrial feed resources for herbivores (forages, cereal-based feeds, crop residues, and browse plants) and their dominant anti-nutritional factors, including tannins, phytates, oxalates, saponins, and alkaloids. Arrows indicate the pathways through which these compounds influence feed intake, digestion, and nutrient utilization.

Terrestrial feed resources constitute the primary dietary components for herbivorous livestock and exhibit substantial variation in botanical origin, nutritional composition, and ANF content [11,27]. These feeds are broadly derived from grasses, legumes, cereal crops, oilseeds, and woody plants, with utilization patterns determined by regional availability, production systems, and species-specific feeding behavior. While terrestrial feeds provide essential energy, protein, fiber, and minerals, they simultaneously represent the principal source of ANFs in herbivore diets, necessitating careful feed selection and processing strategies. Notably, oilseed meals, with soybean meal as the most representative type, are the most economically valuable and dominant protein feed materials in modern herbivore breeding systems [28]. Given their high practical application rate and great significance in industrial production, this chapter focuses on systematically elaborating the composition, functional mechanisms, and mitigation strategies of anti-nutritional factors in oilseed meals, while providing generalized overview descriptions of forage and cereal feeds [29]. The main terrestrial feed resources and their dominant anti-nutritional factors, together with the pathways affecting feed intake and digestion, are illustrated in Figure 1.

### 3.1. Forages and Pasture-Based Feeds for Herbivores

Forages, encompassing both grasses and legumes, form the nutritional foundation of herbivore production systems, particularly in grazing-based and forage-dependent operations [30,31]. Common forage grasses—including *Lolium perenne* (ryegrass), *Phleum pratense* (timothy), and *Zea mays* (maize silage)are generally characterized by moderate ANF concentrations, whereas leguminous forages such as *Medicago sativa* (alfalfa), *Trifolium* spp. (clover), and *Vicia* spp. (vetch) typically contain elevated levels of tannins, saponins, and protease inhibitors [32,33,34,35,36]. In addition, legumes contain amino acid analog anti-nutritional factors, typically canavanine in *Vicia ervilia* (bitter vetch), a non-protein amino acid analogous to arginine. Canavanine interferes with normal protein synthesis and induces the formation of aberrant proteins, thereby reducing meat protein and ash contents and impairing animal performance. It is a key anti-nutritional factor limiting the utilization of bitter vetch in herbivore diets [37,38]. These compounds exert multifaceted effects on ruminant nutrition, influencing ruminal protein degradability, mineral bioavailability, and voluntary feed intake [39]. The concentration and biological activity of ANFs in forages are strongly influenced by plant maturity stage, seasonal variation, soil fertility status, and environmental stress conditions including drought, temperature extremes, and nutrient deficiency. 

### 3.2. Cereal Grains, Crop Residues, and Agro-Industrial By-Products

Cereal grains and their processing by-products (maize/corn, wheat, barley, sorghum) are extensively utilized in herbivore diets as concentrate ingredients to enhance dietary energy density, while oilseed meals (soybean meal, canola/rapeseed meal, cottonseed meal, sunflower meal) are incorporated to increase metabolizable protein supply and improve amino acid balance [40]. Various agro-industrial by-products such as wheat bran, rice bran and soybean residues are widely used in practical herbivore breeding, which are low-cost and cost-effective feed resources. Wheat bran contains moderate crude fiber and crude protein and abundant B vitamins. With soft texture and good palatability, it can regulate gastrointestinal function and promote intestinal peristalsis, and is often added to daily diets of donkeys, cattle and sheep to reduce breeding costs. Rich in fat, vitamins and high in energy, rice bran can improve hair condition and physical health of herbivores, which is mainly used in the fattening period. Its feeding amount should be properly controlled to prevent intestinal discomfort, poor appetite and diarrhea. Soybean residue is rich in plant protein with high cost performance and good palatability when fed fresh, which can effectively supplement dietary protein. Moderate dosage is required to avoid flatulence and indigestion that affect animal feed intake. However, these feed materials frequently contain substantial concentrations of phytates, non-starch polysaccharides, protease inhibitors, and phenolic compounds that can impair nutrient digestibility, reduce mineral absorption, and compromise animal performance [41].

#### 3.2.1. Oilseed Meals as Plant Protein Feeds and Associated Anti-Nutritional Factors

Oilseed meals—obtained following mechanical or solvent extraction of oil from crops such as soybean, rapeseed /canola, cottonseed, and sunflower—represent the most widely utilized plant-derived protein concentrates in herbivore nutrition globally. Soybean meal (SBM), rapeseed meal (RSM), and cottonseed meal (CSM) are particularly valued for their high crude protein content (typically 40–50% dry matter basis), favorable amino acid profiles, and widespread commercial availability, rendering them indispensable components of both ruminant and non-ruminant herbivore feeding systems [42,43].

Despite their considerable nutritional advantages, oilseed meals contain diverse anti-nutritional factors that can compromise nutrient utilization, impair gut health, and reduce animal performance [44]. Common ANFs present in oilseed meals include protease inhibitors (trypsin and chymotrypsin inhibitors), antigenic proteins, lectins (hemagglutinins), phytic acid (phytate), non-starch polysaccharides, gossypol (cottonseed-specific), and glucosinolates (rapeseed/canola-specific). The type, concentration, and physicochemical properties of these compounds differ markedly among feed sources and are influenced by cultivar genetics, growing conditions, and processing methods, necessitating feed-specific characterization and mitigation strategies [45].

#### 3.2.2. Soybean Meal–Related ANFs

Soybean meal represents the most extensively used plant protein feed globally, owing to its high protein digestibility, balanced amino acid composition, and consistent supply [43]. However, raw and inadequately processed SBM contains several biologically active ANFs, including antigenic proteins (β-conglycinin and glycinin), trypsin inhibitors (Bowman–Birk and Kunitz types), soybean agglutinin (lectin), tannins, phytic acid, and non-starch polysaccharides.

Antigenic proteins, primarily β-conglycinin (7S globulin) and glycinin (11S globulin), constitute the major storage proteins in soybeans and can elicit immune-mediated hypersensitivity reactions, particularly in young animals with immature immune systems [46,47]. These proteins can trigger intestinal inflammation, villus atrophy, and reduced growth performance. Trypsin inhibitors—predominantly Bowman–Birk inhibitor (BBI) and Kunitz trypsin inhibitor (KTI)—interfere with pancreatic proteases (trypsin and chymotrypsin), reducing protein digestibility and inducing compensatory pancreatic hypertrophy. Although present in relatively modest quantities (1.5–3.0% of seed weight), their biological impact is substantial, accounting for approximately 30–50% of soybean’s total anti-nutritional activity [48].

Soybean agglutinin (SBA) is a heat-stable lectin with hemagglutinating properties that binds specifically to N-acetyl-D-galactosamine and galactose residues on intestinal epithelial glycoproteins, disrupting brush border membrane integrity, impairing nutrient absorption, and compromising gut barrier function [49,50]. While ruminal microorganisms can partially degrade SBA through proteolytic activity, its adverse effects remain more pronounced in young ruminants with underdeveloped rumens and in non-ruminant herbivores lacking extensive microbial fermentation capacity [51,52].

Additional ANF components—including condensed tannins, phytic acid, and non-starch polysaccharides (arabinoxylans, pectins)—further constrain nutrient availability through multiple mechanisms: forming insoluble complexes with dietary proteins and endogenous enzymes, chelating essential minerals (particularly iron, zinc, calcium, and magnesium), and increasing digesta viscosity, thereby reducing nutrient-enzyme contact and absorption efficiency [53,54,55,56,57,58,59,60].

#### 3.2.3. Cottonseed and Rapeseed Meal–Related ANFs

Cottonseed meal is uniquely characterized by the presence of gossypol, a polyphenolic aldehyde compound that exists in free and bound forms, collectively referred to as total gossypol [61,62]. Free gossypol is biologically active and can damage gastrointestinal tissues and impair reproductive function, particularly in males. Although ruminants exhibit greater tolerance due to rumen detoxification mechanisms, prolonged exposure may still negatively affect fertility [62,63,64]. The World Health Organization has recommended a maximum threshold of 450 mg/kg for free gossypol in cottonseed protein products [65]. Notably, this threshold was originally established for human food safety rather than animal feeds, and herbivores exhibit species-specific tolerance to free gossypol. Rapeseed meal contains glucosinolates that are enzymatically hydrolyzed into toxic metabolites such as oxazolidinethione, isothiocyanates, and nitriles. These compounds primarily affect thyroid function and may also damage the gastrointestinal tract, liver, and kidneys [66,67,68]. Tolerance to glucosinolates varies among animal species, with ruminants generally exhibiting higher resistance than pigs and poultry [69,70]. The main forage species consumed by herbivores and their associated anti-nutritional factors are summarized in Table 1. It lists common forage types, representative species, major anti-nutritional factors, and potential nutritional effects.

Having established where anti-nutritional factors occur across these feed resources, the analysis now shifts from their distribution to their function. The following section examines the biochemical, digestive, microbial, and metabolic pathways through which these compounds act within the herbivore, providing the mechanistic basis required to interpret the performance and health outcomes discussed thereafter.

## 4. Mechanisms of Action of Anti-Nutritional Factors in Herbivores

Anti-nutritional factors exert their effects through multiple interconnected biochemical, physiological, and microbial pathways that ultimately influence feed intake, nutrient digestion, absorption, nutrient utilization, and animal performance [79,80]. These mechanisms are often interdependent and depend on the chemical structure and properties of the ANF, its dietary concentration, and the digestive physiology of the herbivore species. Understanding these mechanisms is critical for interpreting the variable responses observed among species and feeding systems and for developing targeted mitigation strategies [81]. Mechanistic pathways of major anti-nutritional factors present in plant protein feeds and their associated physiological effects in herbivores are summarized in Table 2. Importantly, the mechanisms described below are interpreted not only at the level of individual enzymes and nutrients but also through recent metabolomic and metagenomic evidence, which links anti-nutritional factor exposure to defined shifts in rumen metabolites (e.g., ammonia-nitrogen, branched-chain fatty acids, and tannin-associated amines), in microbial community structure, and in downstream host metabolic pathways such as nitrogen and energy metabolism [82].

### 4.1. Effects on Feed Intake and Palatability

Many anti-nutritional factors directly affect voluntary feed intake by altering palatability through sensory and post-ingestive mechanisms [103]. Compounds such as tannins, saponins, and alkaloids impart bitter, astringent, or off-flavors, leading to reduced voluntary feed intake and avoidance behavior [103,104,105]. These plant secondary metabolites may also influence voluntary feed intake by altering sensory attributes and post-ingestive responses through interactions with chemoreceptors and gut–brain signaling pathways [106]. Condensed tannins reduce palatability through interactions with salivary proteins, forming insoluble complexes that produce astringent sensations, which can suppress intake and nutrient consumption [107,108].

In grazing systems, herbivores often exhibit selective grazing behavior, reducing intake of or actively avoiding plant species and specific plant parts with elevated concentrations of anti-nutritional compounds, resulting in shifted grazing patterns and altered nutrient intake profiles [109,110,111]. Prolonged exposure to unpalatable feeds can result in reduced energy intake, compromised growth, impaired immune function, and poor productive performance.

### 4.2. Interactions with Digestive Enzymes and Nutrients

A major mechanism by which ANFs impair nutrition is through inhibition of key digestive enzymes and reduction in nutrient availability/digestibility through formation of insoluble complexation or binding complexes of nutrients [77,112,113]. Protease inhibitors, particularly trypsin inhibitors, bind to the active sites of pancreatic proteolytic enzymes such as trypsin and chymotrypsin, thereby reducing protein hydrolysis and amino-acid absorption [114]. Two commonly characterized classes include Kunitz inhibitors (primarily targeting trypsin) and Bowman–Birk inhibitors (inhibiting both trypsin and chymotrypsin) [115].

Similarly, Phytic acid chelates divalent and trivalent cations, forming insoluble complexes with proteins and minerals, thereby limiting their bioavailability and increasing endogenous nutrient losses [116,117]. Phytic acid is the principal storage form of phosphorus in many plant seeds and brans and can reduce nutrient utilization by binding minerals (particularly Ca, Mg, Zn, Fe) and interacting with dietary proteins and carbohydrate macromolecules, thereby decreasing digestibility [77,78]. In non-ruminants, which lack sufficient endogenous phytase activity, phytate is poorly degraded and may contribute to reduced growth performance and increased phosphorus excretion, creating both nutritional and environmental concerns [78]. Meanwhile, excessive anti-nutritional factors, particularly high tannin levels, inhibit rumen microbial proteolysis, disrupt nitrogen metabolism, and impair urea cycle function, accompanied by reduced blood urea and ammonia-nitrogen levels, which collectively trigger nitrogen metabolic disorders [118]. Battelli et al. confirmed via an in vitro experiment that high levels of condensed and hydrolysable tannins significantly reduce ruminal ammonia-nitrogen by 17–18%, accompanied by decreased concentrations of branched-chain volatile fatty acids and inhibited dry matter digestibility. These substances also alter the rumen microbial community structure to some extent, decreasing fibrolytic bacterial populations and increasing the relative abundance of *Prevotella*, thereby exacerbating impaired proteolysis and nitrogen metabolic disorders [119]. Furthermore, they alter short-chain fatty acid profiles and suppress tricarboxylic acid cycle activity, ultimately resulting in oxidative stress and energy imbalance [118].

### 4.3. Effects on Rumen Fermentation and Gut Microbiota

Anti-nutritional factors—particularly plant secondary metabolites such as tannins and saponins—can modulate rumen microbial communities (bacteria, protozoa, fungi, and methanogenic archaea) and thereby alter rumen fermentation characteristics including volatile fatty acid profiles, methane production, and microbial protein synthesis [53,96]. Saponins act as natural rumen modifiers by altering the composition and activity of rumen microbiota, including bacteria, protozoa, fungi, and methanogenic archaea through membrane-disrupting effects and selective antimicrobial activity. These microbial shifts subsequently influence fermentation end-products such as volatile fatty acids, ammonia-N, and microbial protein synthesis, thereby affecting nutrient utilization and overall animal performance [120,121,122]. Condensed tannins can bind to dietary proteins and microbial enzymes, reducing ruminal protein degradation and shifting nitrogen metabolism. At moderate concentrations, this protein-binding effect may beneficially increase bypass protein supply to the small intestine; however, excessive tannin levels can impair fiber digestion and reduce overall nutrient utilization [123]. The effects of tannins on rumen fermentation are concentration-dependent and influenced by tannin structure, molecular weight, and dietary context. Studying wildlife, Menke et al. also confirmed that plant secondary metabolites in forage significantly influence the physiological status and gut microbial composition of moose. This shows that species-specific tolerance to anti-nutritional factors is fundamentally linked to gut microbial communities. Regarding tannins—a common anti-nutritional factor—Sun et al. reported that chestnut tannins inhibit the activity of cellulolytic bacteria in the sheep rumen, slowing down fiber digestion. This directly links anti-nutritional factors to rumen microbial function [124,125].

Beyond these community-level shifts, the rumen microbiome acts as an active detoxification compartment whose capacity is itself shaped by anti-nutritional factor exposure [126]. Repeated dietary exposure can enrich tannin- and saponin-tolerant taxa and select for microbial enzymes (e.g., tannases and related hydrolases) that cleave or transform these compounds, so that the same dietary factor elicits markedly different responses depending on the animal’s prior adaptation. This microbial transformation generates intermediate and end-product metabolites—such as altered volatile fatty acid ratios, reduced ammonia-nitrogen, and modified amine profiles—that are subsequently absorbed and feed directly into host nitrogen, energy, and antioxidant metabolism [127]. Viewing rumen detoxification as a dynamic microbiome–metabolite–host axis, rather than a fixed species trait, helps explain the dose-dependent and species-specific tolerance summarized above and identifies the microbial metabolite pool as a tractable target for metabolomics-guided feed evaluation [128].

**Figure 2:**
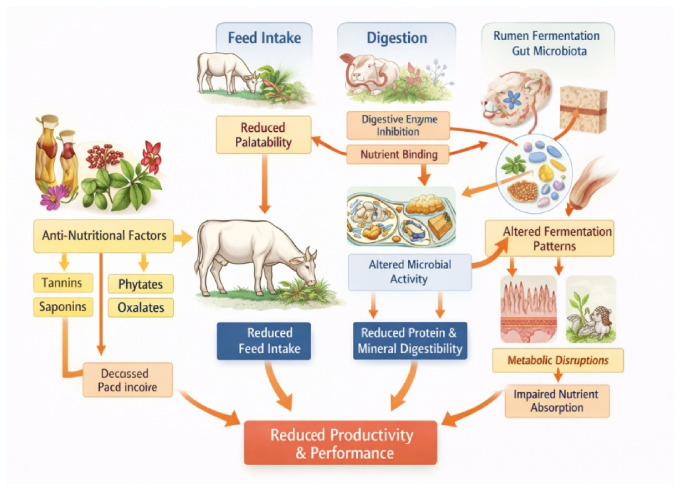
Conceptual illustration of the multifaceted pathways through which plant anti-nutritional factors influence herbivore productivity. ANFs reduce feed intake via palatability effects, impair digestion through enzyme inhibition and nutrient binding, and modulate rumen microbiota and fermentation patterns, collectively resulting in reduced nutrient utilization and animal performance.

Overall, anti-nutritional factors influence animal performance through integrated pathways involving reduced feed intake, impaired digestion, altered rumen microbiota and fermentation, and decreased nutrient absorption, culminating in reduced productivity, as summarized in Figure 2.

These molecular and microbial mechanisms do not operate in isolation; their combined effects converge at the level of the whole animal. The following section therefore evaluates how anti-nutritional factors ultimately shape feed intake, growth, reproduction, and health, and how the same mechanisms underlie the dose-dependent, dual effects observed across different herbivore species [129].

## 5. Effects of Anti-Nutritional Factors on Herbivore Performance and Health

Anti-nutritional factors influence herbivore productivity through their cumulative effects on feed intake, nutrient digestion, absorption, nutrient utilization, and metabolic regulation [14,130]. Across herbivore production systems, ANFs can reduce feed intake, impair nutrient digestibility, and decrease conversion efficiency, and may impair organ function when exposure is high or prolonged, largely through reduced digestibility, nutrient binding, and intestinal or metabolic disruption [14,130]. The severity of these effects depends on the type and concentration of ANFs, diet composition, animal species and physiological state, duration of exposure, and the physiological characteristics of the animal. While acute toxicity may occur at high intake levels, more commonly ANFs exert subclinical effects that gradually reduce performance and compromise animal health. Notably, ANFs exhibit dose-dependent dual effects: excessive levels are harmful, while appropriate concentrations can confer beneficial functions such as regulating gut microbiota and antioxidant protection. At appropriate doses, tannins protect dietary protein from excessive ruminal degradation, increasing post-ruminal protein and amino acid absorption, thereby enhancing nitrogen retention and reducing urinary nitrogen loss [118].

However, recent studies have shown that tannins, as traditional anti-nutritional factors, exert positive effects on animal production and product quality when administered at appropriate doses [131]. A meta-analysis of 97 studies indicated that dietary tannins exert beneficial effects on the growth performance of small ruminants, significantly improving nitrogen metabolism and reducing urinary nitrogen excretion; meanwhile, they enhance the fatty acid profile of meat by lowering the proportion of saturated fatty acids, thereby improving meat quality [118]. Consistent with these findings, feeding trials further confirmed that tannin-rich Sulla flexuosa hay can replace alfalfa in goat diets. Dietary inclusion of 35% and 70% (dry matter basis) Sulla flexuosa hay has no adverse effects on goat milk yield or routine milk composition; notably, the 70% inclusion group significantly increases the contents of monounsaturated fatty acids (MUFA), polyunsaturated fatty acids (PUFA) and docosahexaenoic acid (DHA) in milk, enhances antioxidant capacity, and reduces the atherogenic index, demonstrating that tannins at appropriate doses can improve milk quality [132].

The primary mechanisms through which ANFs impair herbivore performance include: (1) reduced voluntary feed intake due to decreased palatability and post-ingestive feedback; (2) impaired protein and energy digestibility through enzyme inhibition and nutrient complexation; (3) reduced mineral bioavailability, particularly phosphorus, calcium, zinc, and iron; (4) altered rumen fermentation patterns and microbial protein synthesis; (5) compromised intestinal integrity and barrier function; and (6) increased metabolic costs associated with detoxification and compensatory physiological responses. These effects manifest as reduced growth rates, decreased milk production, impaired reproductive performance, and compromised immune function, with economic implications for production efficiency and profitability.

Anti-nutritional factors are chemicals that have evolved in plants for their own defense, among other biological functions and reduce the maximum utilization of nutrients, especially proteins, vitamins, and minerals, thus preventing optimal exploitation of the nutrients present in a food and decreasing the nutritive value [133].

In addition to general ruminant vs. non-ruminant differences, herbivores exhibit distinct ANF tolerance based on feeding strategy (grazers, browsers, intermediates), as well as age and physiological status. These species-specific and physiological variations are summarized in Table 3.

To provide clear feed safety references and practical guidelines for strategic ANF management, the toxicity thresholds, optimal beneficial levels, and species-specific tolerance limits of major anti-nutritional factors are summarized in Table 4.

Given these performance and health consequences, attention now turns from characterizing the problem to managing it. The following section critically assesses the physical, chemical, biological, and genetic strategies available for mitigating anti-nutritional factors, evaluating each with respect to efficacy, scalability, nutrient preservation, and sustainability.

## 6. Strategies for Mitigating Anti-Nutritional Factors in Terrestrial Feeds

**Figure 3:**
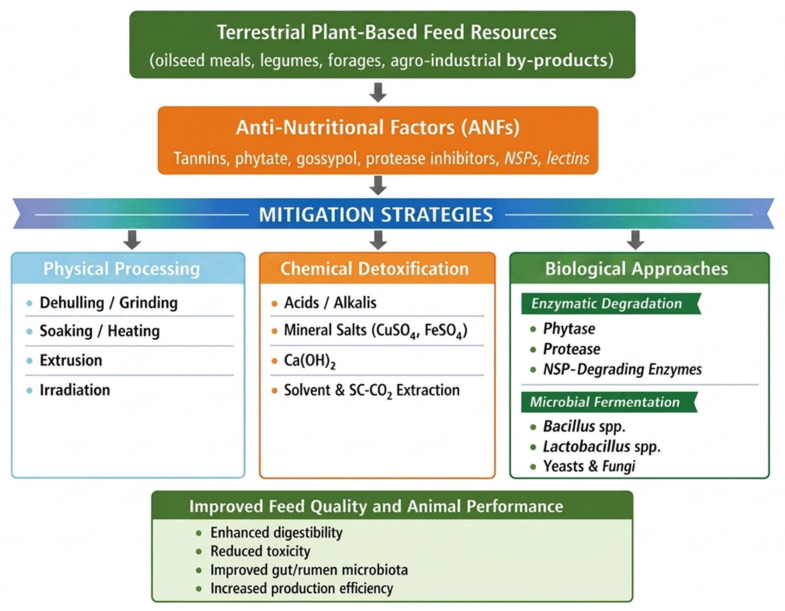
Schematic overview of strategies for controlling anti-nutritional factors (ANFs) in herbivore diets. Mitigation approaches are grouped into physical processing, chemical detoxification, and biological methods (enzymes and microbial fermentation), which collectively improve feed quality, nutrient utilization, and animal performance.

Effective management of anti-nutritional factors is essential to maximize nutrient utilization, animal performance, and feed efficiency in herbivore production systems. Mitigation strategies aim not only to reduce ANF concentrations but also to minimize their biological activity and negative interactions with nutrients [95,135]. The selection of an appropriate method depends on the type and thermal stability of the ANF, its stability during processing, the animal species being fed, its digestive physiology, and the balance between detoxification efficiency and nutrient preservation [136]. These strategies can be broadly categorized into physical, chemical, biological, and genetic approaches, often used in combination to achieve optimal results. In addition to physical, chemical, and biological detoxification technologies, precision nutritional regulation can synergistically improve feed utilization efficiency [137]. Figure 3 systematically summarizes the overall framework of ANF control technologies for herbivore diet.

### 6.1. Physical Processing Methods

Physical processing techniques are widely applied due to their simplicity, practical feasibility, and cost-effectiveness. Methods such as grinding, soaking, pelleting, and heat treatment can reduce the activity of certain ANFs, particularly enzyme inhibitors and some phenolic compounds [138,139,140].

#### 6.1.1. Thermal Methods

These mechanical approaches are effective for reducing tannins and phytate-associated compound fractions, although efficacy varies considerably across plant species and seed anatomical structures. Heat treatment remains the predominant strategy for inactivating thermolabile ANFs, including many protease inhibitors and lectin-type proteins [141]. Both dry heating methods (e.g., roasting, microwave exposure, infrared heating) and moist heating techniques (e.g., boiling, autoclaving, extrusion) are widely employed [142]. Similarly, thermal treatments including boiling, cooking, and autoclaving are effective in reducing heat-labile ANFs such as protease inhibitors and lectins, thereby enhancing feed digestibility and nutritional quality [72,143]. Among these, autoclaving has been reported to be particularly efficient due to the combined effects of heat and pressure in denaturing anti-nutritional compounds [144,145].

#### 6.1.2. Mechanical Methods

Thermal processing, including roasting, autoclaving, and steam treatment, is effective in denaturing protease inhibitors present in oilseed meals and inactivating heat-labile lectins [146]. Physical processing methods aim to reduce ANF concentrations through mechanical removal, structural disruption, or heat-induced inactivation. Dehulling, peeling, crushing, and grinding can effectively lower ANF levels when these compounds are preferentially concentrated in outer seed layers or specific tissue fractions [147]. Milling is another widely employed method that reduces ANF content by removing outer seed layers where compounds such as tannins, phytates, and lectins are concentrated. However, this process may also result in the loss of essential nutrients, particularly minerals [148]. Milling is the most traditional method to separate the bran layer from the grains. It is a process by which grains are ground into flour [149]. However, milling not only removes anti-nutritional factors that affect digestion and absorption, but also often eliminates minerals and other nutrients in the feed [150].

#### 6.1.3. Soaking

Soaking is one of the most commonly applied techniques, facilitating the removal of water-soluble anti-nutritional factors and activating endogenous enzymes such as phytases, which contribute to the degradation of phytates and improvement of mineral availability [148,151]. Soaking is an attractive method for removing anti-nutritional factor content of foods because it also reduces cooking time. Soaking also enhances release of enzymes (e.g., endogenous phytases), which are present in plant foods like almonds and other nuts and grains [152]. Similarly, soaking also reduces the nutrient content in feed. Studies have shown that soaking followed by autoclaving reduces free gossypol and phytate concentrations in oilseed meals, while water immersion helps remove soluble non-starch polysaccharides [152,153]. Beyond these qualitative observations, quantitative trade-offs exist: soaking can reduce phytic acid by 20–40% depending on temperature and duration, but also leads to measurable losses of water-soluble vitamins (e.g., B-complex) and minerals such as K and Mg [95,143]. The net nutritional outcome depends on soaking conditions (time, temperature, grain-to-water ratio) and whether the soaking water is discarded.

#### 6.1.4. Extrusion/Irradiation

Extrusion processing integrates thermal energy, mechanical pressure, and shear forces to effectively reduce enzyme inhibitors such as trypsin inhibitors and soybean agglutinin while maintaining protein integrity [154]. The efficiency of extrusion is strongly influenced by processing parameters, including moisture content, barrel temperature, screw speed, and feed rate [155]. Comparative studies indicate that although extrusion can substantially reduce phytate levels, biological processes such as germination may achieve greater reductions in certain legume species [156]. In addition, irradiation technologies including electron beam and gamma irradiation have shown dose-dependent efficacy in reducing free and total gossypol content in cottonseed meal, with electron beam treatment generally exhibiting superior efficiency [157,158].

Therefore, physical processing alone is often insufficient to eliminate chemically stable compounds such as phytates and oxalates and may result in nutrient losses including heat-sensitive vitamins and amino acids if not properly managed [159].

### 6.2. Chemical Treatments

Chemical methods are used to alter the chemical structure or reactivity of anti-nutritional factors through hydrolysis, oxidation, or complexation reactions. Alkali treatments, such as ammonia or sodium hydroxide application, can reduce tannin activity and improve fiber digestibility in low-quality roughages by disrupting lignin-carbohydrate complexes. Acid treatments and mineral supplementation may partially counteract the effects of mineral-binding ANFs by improving nutrient solubility and disrupting phytate-mineral complexes [160,161,162,163]. Supercritical carbon dioxide (SC-CO_2_) extraction of cottonseed oil (pressure > 550 bar, 70–80 °C, 2–3 h) has also been reported to markedly reduce gossypol content, although its application remains constrained by current processing technology [164,165].

Chemical methods involve the use of alkalis, organic solvents, or binding agents to modify or inactivate anti-nutritional compounds. For example, polyethylene glycol (PEG) has been widely used to bind tannins and prevent their interaction with dietary proteins, thereby improving nutrient availability [164,165]. Similarly, alkaline treatments can reduce phenolic compounds and improve feed digestibility. Low-cost alternatives such as wood ash have also been traditionally used to reduce tannin content in feed materials, highlighting the potential of simple chemical approaches in practical feeding systems [164,165].

While chemical treatments can be effective, their use is often constrained by cost, safety concerns, potential residual chemical contamination, environmental impact, and regulatory limitations. However, the application of chemical treatments must be carefully managed due to potential safety, environmental, and regulatory concerns [166].

### 6.3. Biological and Microbial Approaches

Biological strategies have gained increasing attention due to their sustainability, environmental compatibility, and specificity. Fermentation and ensiling promote microbial degradation of certain ANFs, including phytates and protease inhibitors, while improving feed palatability and generating beneficial metabolites. Enzyme supplementation, such as phytase addition, enhances mineral bioavailability by hydrolyzing phytate complexes and releasing bound phosphorus [149,167,168].

Probiotics and rumen modifiers can support microbial populations capable of detoxifying ANFs, thereby improving rumen fermentation efficiency and nutrient utilization [169,170].

Germination treatment of feed represents an important approach to enhance nutritional value, reduce anti-nutritional factors, and improve digestibility and absorption by means of biodegradation driven by seed endogenous enzymes [171]. Biological processing techniques have gained increasing attention due to their sustainability and efficiency in reducing ANFs while improving feed quality. Germination activates endogenous enzymes that degrade anti-nutritional compounds and mobilize stored nutrients, resulting in improved digestibility and nutrient availability [172,173]. Significant reductions in tannins and other secondary metabolites have been reported during germination processes in legume seeds [156]. Fermentation is another effective approach in which microbial activity reduces ANFs such as phytates, tannins, and protease inhibitors while enhancing protein digestibility and mineral bioavailability [148,174]. These biological methods are increasingly applied in modern feed systems due to their dual role in detoxification and nutritional enhancement [175].

### 6.4. Plant Breeding and Genetic Improvement

Long-term mitigation of anti-nutritional factors can be achieved through plant breeding, genetic selection, and genomic editing technologies. Development of low-ANF cultivars has been successful for several feed crops, particularly in reducing phytate content in grains, glucosinolate levels in rapeseed/canola, and tannin levels in forage legumes. Advances in molecular breeding, marker-assisted selection, and genomic tools including CRISPR-Cas9 gene editing offer opportunities to target specific biosynthetic pathways responsible for ANF production [176,177]. However, reducing ANF contents must be balanced against plant resistance to pests, diseases, and environmental stress, as many ANFs serve protective functions in plant defense mechanisms. Therefore, in the process of breeding and processing improvement, precise regulation strategies should be adopted [178]. While appropriately reducing ANF levels to enhance nutritional value, the basic defense functions of plants should be preserved. Through the coordinated optimization of variety breeding, moderate processing and cultivation management, a multiple balance among nutritional quality, plant resistance and production stability can be achieved [179].

### 6.5. Integrated Feed Management Approaches

In practice, the most effective mitigation strategies involve integrated feed management rather than reliance on a single method. Combining feed processing, enzyme dietary supplementation, strategic blending of feed ingredients, and controlled inclusion rates allows producers to manage ANF exposure while maintaining feed diversity, nutritional balance, and cost efficiency. Precision feeding approaches, informed by comprehensive feed analysis and animal requirements, further enhance the ability to minimize ANF-related risks in herbivore diets while optimizing nutrient supply and production outcomes [180]. Future research should focus on the interactions among different anti-nutritional factors, explore integrated regulation strategies of ANFs suitable for various herbivores, and establish scientific and unified risk assessment standards and systems for anti-nutritional factors. These are the key research gaps that urgently need to be filled at present [181].

### 6.6. Sustainable Feed Development and Circular Agriculture

**Figure 4:**
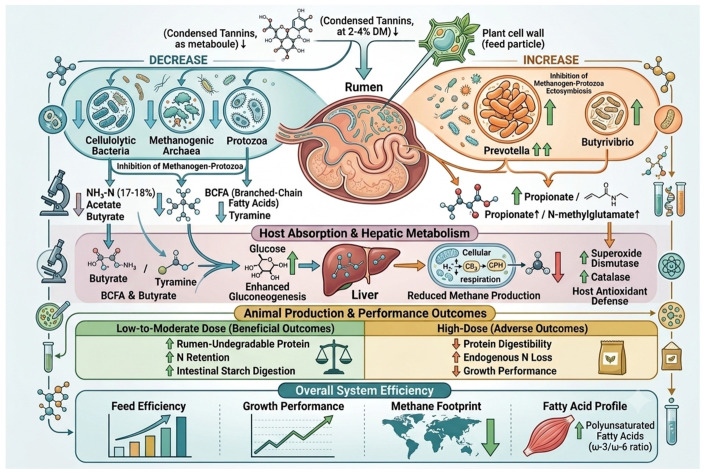
Mechanistic network of condensed tannins in ruminant nutrition and metabolism. The figure presents the dose-dependent effects of condensed tannins (2–4% DM) on rumen microbiota, metabolite shifts, host metabolism, and production/environmental outcomes in herbivores. Moderate tannin inclusion modulates microbial community and fermentation to improve nitrogen efficiency and reduce methane emissions, while high doses induce metabolic disorders. Note: ↑ = Increase, ↓ = Decrease. Blue: Beneficial effects of low-moderate CT (2–4% DM). Orange: Promoted microbes and metabolites. Yellow: Adverse effects of high-dose CT. Abbreviations: ANF: Anti-Nutritional Factor; DM: Dry Matter; BCFA: Branched-Chain Fatty Acids; NH_3_-N: Ammonia Nitrogen; N: nitrogen.

Recent research work emphasizes that some ANFs can exert beneficial effects at appropriate inclusion levels when properly managed. For example, condensed tannins at moderate concentrations (2–4% of dietary dry matter) may improve protein utilization by reducing ruminal protein degradation and increasing bypass protein flow to the small intestine, and contribute to parasite control through direct anthelmintic effects, depending on chemical structure, molecular weight, and dosage [32,182,183]. Likewise, saponins have been investigated as modulators of rumen fermentation, including potential defaunation effects that may improve nitrogen utilization efficiency and reduce methane emissions, although outcomes are diet- and dose-dependent [75,76,120]. These findings highlight the importance of dose–response relationships and suggest that strategic management of certain ANFs may offer functional benefits beyond simple elimination. For example, condensed tannins specifically function by forming tannin–protein complexes, which reduce ruminal protein degradation and increase bypass protein flow, thereby improving nitrogen utilization efficiency [118]. Mechanistically, tannins decrease the abundance of methanogenic archaea (e.g., Methanobrevibacter) and protozoa, reducing hydrogen availability for methanogenesis and thus lowering methane emissions [183]. Saponins similarly inhibit methanogens and ciliate protozoa, shifting rumen fermentation toward propionate production [104]. Recent metabolomics studies further show that tannin supplementation decreases tyramine (a methane-related metabolite) while increasing N-methylglutamic acid, which is less involved in methanogenesis [183]. These effects are dose-dependent, indicating that strategic management rather than complete elimination of anti-nutritional factors can enhance environmental sustainability while maintaining animal productivity. These microbially mediated metabolite shifts form a dose-dependent regulatory network from microbiota to host. To visualize this mechanism, we constructed the following pathway diagram Figure 4.

Drawing these strands together, the concluding section integrates the compositional, mechanistic, metabolomic, and management perspectives developed above, and outlines the systems-level and precision nutrition priorities most likely to advance anti-nutritional factor management in the coming year.

## 7. Conclusions

Anti-nutritional factors are no longer adequately conceptualized as uniformly detrimental compounds. They act through interconnected pathways—depressed feed intake, enzyme inhibition, nutrient complexation, modulation of rumen microbial ecology, and altered post-absorptive metabolism—whose net effect is governed by ANF class, dose, dietary matrix, animal species, feeding type, age, and physiological state. While ruminants benefit from partial microbial detoxification, prolonged or excessive exposure still compromises growth, reproduction, and health. Conversely, controlled doses of selected plant secondary metabolites improve nitrogen retention, suppress methanogenesis, and confer anthelmintic activity, supporting a dose-dependent management paradigm rather than categorical exclusion.

Current mitigation approaches—physical, chemical, biological, and genetic—each carry intrinsic trade-offs. Physical processing is scalable but causes losses of vitamins, minerals, and heat-labile amino acids. Chemical treatments raise residue, environmental, and regulatory concerns. Conventional fermentation is effective against heat-labile inhibitors and antigenic proteins yet limited against chemically stable compounds such as phytate and oxalate. Low-ANF cultivars offer durable solutions but may erode the plant’s intrinsic defense.

Four converging technologies are reshaping this landscape: (i) green processing—ultrasound-assisted enzymatic hydrolysis, low-temperature plasma, and supercritical CO_2_ extraction—that detoxifies ANFs while preserving nutrients; (ii) designed microbial consortia that colonize the gastrointestinal tract for substrate-specific degradation; (iii) CRISPR-mediated editing of biosynthetic pathways that lowers ANF content without dismantling plant defense; and (iv) AI-driven formulation that dynamically optimizes inclusion rates against species, physiology, and feed composition.

Future research should advance along four pillars. Metabolomics-guided feed evaluation will replace static compositional analysis with mechanistically informed risk–benefit profiling based on rumen and circulating metabolite signatures. Precision feeding, informed by individual microbiome and metabolic phenotyping, will translate species-average guidelines into animal-specific dosing windows. Biomarker discovery should target early indicators of subclinical ANF exposure to enable proactive intervention. Systems-biology integration across plant chemistry, microbial transformation, and host physiology will be essential to predict multi-ANF, mixed-ingredient diets under climatic and resource stress.

Collectively, the evidence supports a shift from eliminating anti-nutritional factors to strategically managing them—an interdisciplinary agenda spanning plant breeding, feed biochemistry, rumen microbiology, omics, and precision livestock nutrition, and central to building safe, productive, and environmentally sustainable herbivore feeding systems.

## Figures and Tables

**Table 1:** Major forage species used in herbivore feeding and their associated anti-nutritional factors.

Forage Type	Representative Species	Major Anti-Nutritional Factors	Potential Nutritional Effects	References
Forage grasses/pasture	Ryegrass (*Lolium perenne*), timothy (*Phleum pratense*), maize forage/silage (*Zea mays*); tropical grasses	Nitrates (stress-related), oxalates (in some grasses)	Reduced intake/performance under high nitrate; nitrite formation can impair oxygen transport; oxalates can reduce Ca availability and disturb Ca: P balance, with severity depending on species and adaptation	[14,71,72,73]
Grasses vs. Legumes	Grasses: Ryegrass (*Lolium perenne*), Timothy (*Phleum pratense*), Maize silage (*Zea mays*) Legumes: Alfalfa (*Medicago sativa*), Clover (*Trifolium* spp.), Vetch (*Vicia* spp.)	Grasses: Generally moderate ANFs; occasional nitrates, endophyte alkaloids Legumes: Tannins, saponins, protease inhibitors, phytoestrogens (species-dependent)	Grasses: Usually, good digestibility with lower ANF interference Legumes: May alter rumen protein degradability, mineral availability, intake, and reproductive performance at high levels	[27]
Legume forages	Alfalfa *(Medicago sativa*), clovers (*Trifolium* spp.), vetch (*Vicia* spp.)	Tannins, saponins, protease inhibitors, phytoestrogens (species-dependent)	Altered rumen protein degradability; reduced digestibility and intake at high tannin; potential rumen fermentation modulation (some benefits at controlled levels)	[32,74,75,76]
Cereal grains	maize (*Zea mays*), wheat (*Triticum aestivum*), barley (*Hordeum vulgare*), rice (*Oryza sativa*)	Phytic acid (phytate), protease inhibitors (some), mycotoxins (storage/field), lectins (lower than legumes)	Mineral chelation and reduced nutrient availability (especially in non-ruminants); performance losses when contaminated by mycotoxins; effects depend on processing and inclusion rate	[16,77,78]

**Table 2:** Mechanistic pathways of major anti-nutritional factors in plant protein feeds and their physiological impacts in herbivores.

Major Feed Sources	Anti-Nutritional Factor	Primary Biochemical Mode of Action	Digestive and Physiological Consequences	References
Soybean, legumes, oilseed meals	Protease inhibitors (Kunitz, Bowman–Birk)	Formation of stable complexes with digestive proteases (trypsin, chymotrypsin), reducing the enzymatic hydrolysis of dietary proteins	Decreased protein digestibility, pancreatic hypertrophy, reduced growth rate, impaired nutrient utilization	[83,84]
Soybean meal	Soybean antigenic proteins (β-conglycinin, glycinin)	Translocation across intestinal epithelium; activation of humoral immune responses and B-cell antibody production; disruption of epithelial tight junctions	Intestinal hypersensitivity, mucosal inflammation, impaired barrier integrity, diarrhea, reduced growth performance	[85,86,87,88,89]
Soybean meal	Soybean agglutinin (SBA)	Lectin binding to intestinal epithelial glycoproteins; modulation of mucosal immunity; inhibition of IgA secretion; induction of epithelial apoptosis via FAK signalling pathways	Impaired nutrient absorption, altered gut microbiota, intestinal epithelial damage, and immune dysregulation	[90,91]
Cottonseed meal	Free gossypol	Binding with amino acids (especially lysine) and metal ions; interference with enzymatic systems; disruption of cellular membranes and cardiac muscle function	Reduced lysine bioavailability, cardiotoxicity, reproductive impairment, anemia, and growth depression	[61,92,93,94]
Oilseed meals, cereals, legumes	Phytic acid (phytate)	Chelation of divalent and multivalent minerals (Zn, Ca, Fe, Cu); inhibition of digestive enzymes, including proteases and amylases	Reduced mineral bioavailability, impaired protein digestion, decreased nutrient absorption efficiency	[95,96,97]
Legumes, browse plants, tree leaves	Tannins	Complex formation with dietary proteins, digestive enzymes, and salivary glycoproteins; interference with mucosal transport processes	Reduced palatability, decreased feed intake, lower protein availability, altered rumen fermentation; potential reproductive metabolic effects at high levels	[98,99,100]
Rapeseed meal, cruciferous feeds	Glucosinolates	Enzymatic hydrolysis into goitrogenic metabolites (thiocyanates, isothiocyanates, oxazolidinethiones) affecting endocrine pathways	Thyroid dysfunction, metabolic disturbances, reduced growth and feed efficiency	[68,101]
Cereal by-products, oilseed meals	Non-starch polysaccharides (NSPs)	Resistance to endogenous enzymatic degradation; increased digesta viscosity; limited fermentability of β-1,4-linked polysaccharides	Reduced nutrient diffusion, impaired digestion, altered rumen fermentation kinetics	[59,60,102]

**Table 3:** ANF tolerance of herbivores by feeding type, age, and physiological status.

Feeding Type	Main ANF Tolerance	Age/Physiology Effects	References
Grazers (e.g., cattle, sheep)	Moderate tolerance to tannins; sensitive to high oxalates and glucosinolates	Young animals: higher sensitivity; mature animals: greater tolerance	[14,27,93]
Browsers (e.g., goats, deer)	High tolerance to tannins and plant secondary metabolites	Lactation/growth: increased susceptibility to ANFs	[13,33,134]
Intermediates (e.g., donkeys, camels)	Broad tolerance to most ANFs; adaptable to mixed diets	Poor body condition: reduced ANF tolerance	[14,27]

**Table 4:** Toxicity thresholds, species-specific tolerance, and optimal beneficial levels of major anti-nutritional factors (dry matter basis).

ANF Type	Ruminants	Non-Ruminants	Optimal Beneficial Level	References
Condensed tannins	Toxic >5.0%	Toxic >1.5%	2.0–4.0%	[118]
Phytate	Toxic >3.0%	Toxic >1.0%	<1.0%	[78]
Saponins	Toxic >3.0%	Toxic >1.5%	1.0–2.0%	[104]
Glucosinolates	<2.5 μmol/g	<1.0 μmol/g	<1.5 μmol	[68,69,70]
Free gossypol	<450 mg/kg	<100 mg/kg	<200 mg/kg	[65]

## Data Availability

No new data were created or analyzed in this study.
